# Boosting Psychotherapy With Noninvasive Brain Stimulation: The Whys and Wherefores of Modulating Neural Plasticity to Promote Therapeutic Change

**DOI:** 10.1155/np/7853199

**Published:** 2024-12-18

**Authors:** Daniele Saccenti, Leonor J. Romero Lauro, Sofia A. Crespi, Andrea S. Moro, Alessandra Vergallito, Regina Gregori Grgič, Novella Pretti, Jacopo Lamanna, Mattia Ferro

**Affiliations:** ^1^Department of Psychology, Sigmund Freud University, Milan, Italy; ^2^Department of Psychology and NeuroMi, University of Milano-Bicocca, Milan, Italy; ^3^Cognitive Studies, Cognitive Psychotherapy School and Research Center, Milan, Italy; ^4^Faculty of Psychology, Vita-Salute San Raffaele University, Milan, Italy; ^5^Center for Behavioral Neuroscience and Communication (BNC), Vita-Salute San Raffaele University, Milan, Italy; ^6^Clinical Psychology Center, Division of Neurology, Galliera Hospital, Genoa, Italy

**Keywords:** combined approach, neural plasticity, noninvasive brain stimulation, psychotherapy, transcranial direct current stimulation, transcranial magnetic stimulation

## Abstract

The phenomenon of neural plasticity pertains to the intrinsic capacity of neurons to undergo structural and functional reconfiguration through learning and experiential interaction with the environment. These changes could manifest themselves not only as a consequence of various life experiences but also following therapeutic interventions, including the application of noninvasive brain stimulation (NIBS) and psychotherapy. As standalone therapies, both NIBS and psychotherapy have demonstrated their efficacy in the amelioration of psychiatric disorders' symptoms, with a certain variability in terms of effect sizes and duration. Consequently, scholars suggested the convenience of integrating the two interventions into a multimodal treatment to boost and prolong the therapeutic outcomes. Such an approach is still in its infancy, and the physiological underpinnings substantiating the effectiveness and utility of combined interventions are still to be clarified. Therefore, this opinion paper aims to provide a theoretical framework consisting of compelling arguments as to why adding NIBS to psychotherapy can promote therapeutic change. Namely, we will discuss the physiological effects of the two interventions, thus providing a rationale to explain the potential advantages of a combined approach.

## 1. Introduction

Psychotherapy encompasses several interventions based on therapist–patient interaction aimed at producing cognitive, emotional, relational, and behavioral changes through novel experiences that trigger improvements in the patient's levels of functioning accompanied by a reduction in symptom severity [1]. Such positive modifications are commonly called “therapeutic change” and require (or promote) a sequence of adaptive structural and functional changes in the brain, which fall under the umbrella term of “brain plasticity” [2, 3]. However, even when gold-standard psychotherapeutic interventions are employed, almost half of patients do not experience significant improvements [4], or encounter relapses within a year after a transient remission of their symptoms [5]. Therefore, alternative interventions or combined approaches should be developed to improve therapy outcomes, reduce individual response variability, and enhance effect duration. Previous evidence highlights that combining psychotherapy with pharmacotherapy can be an effective strategy to counter psychiatric disorders' symptoms [6–8]. Nonetheless, drugs present relevant downsides, including a wide range of adverse effects and low compliance [9]. This observation has led researchers to explore other potential treatment strategies, especially for patients with refractory disorders or subjects experiencing intolerable aftermaths linked to medication. Among the currently available interventions, noninvasive brain stimulation (NIBS) emerges as a promising candidate to be combined with psychotherapy. Indeed, NIBS is designed to selectively target distinct brain circuits, causing long-lasting effects on neural excitability and synaptic transmission without sorting unbearable side effects in most cases [10, 11]. Scholars came up with such intuition since both psychotherapy and NIBS, as monotherapies, have proven their effectiveness in ameliorating the symptoms of numerous psychiatric populations [12–14]. Hence, combining the two interventions could have promoted faster and longer lasting improvements in patients' symptoms [15–19]. Another hypothesis is that NIBS enhances psychotherapeutic effects by tailoring the treatment to the patient's unique characteristics and therapeutic goals while allowing more direct control of therapy-induced neuronal changes [15]. However, the physiological rationale behind the effectiveness and usefulness of such integrated treatment is not yet fully understood. Therefore, this opinion paper aims to provide a theoretical framework of compelling arguments for why adding NIBS to psychotherapy can promote therapeutic change.

## 2. Principles of Neural Plasticity

To acknowledge the reasons behind coupling psychotherapy with NIBS, it is crucial to consider the brain as an ever-changing organ, that is, a “*plastic*” entity that constantly reshapes itself throughout its entire lifespan. In this framework, neural plasticity refers to the capacity of the nervous system to modify itself, functionally and structurally, in response to internal or external perturbations [20]. Among the different forms of neural plasticity currently discovered (e.g., spike-timing-dependent [21], anti-Hebbian [22], and homeostatic plasticity [23]), Hebbian plasticity results closely associated with memory and learning [24], and presumably to therapeutic change. Specifically, long-term potentiation (LTP) and long-term depression (LTD) are typical examples of Hebbian mechanisms [25]. These processes, brought to the forefront by the work of Bliss et al. [26–28], entail long-lasting changes in the pattern of synaptic weights, allowing neurons to strengthen, in the case of LTP, or weaken, in the case of LTD, their connections [29]. Such phenomena have been most observed in the hippocampus [30], but they also occur in other brain regions, such as basal ganglia [31], cerebellum [32], neocortex [33], and visual cortex [34]. Hebbian plasticity in the basolateral nucleus of the amygdala, especially, is required for the encoding and maintenance of fear-related memories, as well as for fear extinction [35]. In the most studied associative forms, LTP and LTD have been shown to involve different patterns of activation of N-methyl-D-aspartate (NMDA) receptors, thus leading to the rise of intracellular levels of calcium which in turn activates signaling cascades involving protein kinases/phosphatases [36]. Also, presynaptic changes can support synaptic plasticity expression, driving both sub- and supra-threshold changes in neural transmission [37, 38]. Importantly, both LTP and LTD comprise functional and morphological changes detectable at the synapse level [39, 40].

In particular, LTP in the lateral and basolateral amygdala could be induced by applying high-frequency stimulation to the synaptic afferents in the external capsule [41], which causes a large NMDA-dependent increase in dendritic spine calcium concentrations [42, 43]. Such an increment leads to the activation of intracellular signaling cascades involving numerous protein kinases, such as calcium/calmodulin (CaM)-dependent protein kinase II (CaMKII), that is, a family of multifunctional serine/threonine protein kinases whose activation is known to induce potentiation of synaptic transmission and occlude further LTP phenomena [44, 45]. In turn, the activation of intracellular signaling cascades leads to an increase in the single-channel conductance of synaptic *α*-amino-3-hydroxy-5-methyl-4-isoxazolepropionic acid (AMPA) receptors and, more importantly, promotes the incorporation of additional AMPA receptors into the postsynaptic density [46, 47]. Notably, the novel synaptic AMPA receptors are stabilized through their interaction with so-called “slot proteins,” that is, a group of scaffold proteins capable of trapping AMPA receptors and reducing their lateral mobility [48, 49]. For example, postsynaptic density protein 95 (PSD-95), that is, a membrane-associated guanylate kinase found in the postsynaptic density, regulates the number of AMPA receptors at individual synapses [50]. Indeed, the overexpression of PSD-95 increases synaptic strength and occludes LTP [51], whereas the knockdown of PSD-95 decreases both the surface expression of AMPA receptors and synaptic strength [52]. Moreover, specific genes, whose expression is induced during the early LTP, encode plasticity-related proteins required for LTP maintenance and memory formation [53]. A subset of these plasticity-evoked genes, known as immediate-early genes (IEGs), has been implicated in the aforementioned molecular events because of their rapid and transient responsiveness to synaptic activation [54]. For instance, expression of IEGs such as *egr*-1, *c-fos*, and *Arc* results rapidly upregulated in the hippocampus and lateral amygdala following the neuronal activation associated with behavioral tasks [55, 56]. In parallel, structural changes within the synapse occur, such that the size of the postsynaptic density and dendritic spines are expanded [39]. Accordingly, the induction of LTP by either electrical or chemical stimulation causes an enlargement or increase in the number of dendritic spines [57–59], an increase in the number of bifurcated spines [60, 61], and the formation of spinules from the spine head [62]. These changes, in turn, drive an increase in the size of the presynaptic active zone such that the potentiated synapses are “permanently” enlarged [63]. The abovementioned modifications are possibly mediated by the action of neurotrophins, such as the brain-derived neurotrophic factor (BDNF), that is, activity-dependent modulators of neuronal structure and function known to support differentiation, maturation, and survival of neurons in the mammalian nervous system [64, 65].

Turning to LTD, such a process could be induced in the basolateral amygdala neurons by applying low-frequency stimulation to the lateral nucleus [66], which causes a modest NMDA-dependent increase in dendritic spine calcium concentrations [67, 68]. This leads to a preferential activation of calcium-dependent protein phosphatases instead of kinases [69]. The main signaling cascades include CaM-dependent phosphatase calcineurin, protein phosphatase 1 (PP1), and inhibitor-1, a phosphoprotein inhibiting PP1 until it is dephosphorylated by calcineurin [70]. Postsynaptic inhibition of these phosphatases consistently prevents LTD [71], whereas directly loading hippocampal pyramidal cells with PP1 enhances LTD [72]. In turn, the activation of protein phosphatase cascades leads to the dissociation of AMPA receptors from their molecular scaffolds and promotes their lateral movement to the periphery of the postsynaptic density, where they are endocytosed and potentially degraded [73, 74]. Furthermore, there is evidence that LTD is accompanied by a shrinkage or loss of dendritic spines [75–77], which could be reverted by a potentiation stimulus [78]. These structural changes have been associated with the loss of AMPA receptors [79]. The main hypothesis is that the activity-dependent trafficking of AMPA receptors into and out of synapses during LTP and LTD, respectively, constitutes the first step in the morphological growth or shrinkage of synapses, thus representing a potential mechanism by which bidirectional changes in synaptic strength are maintained [25]. Indeed, the size of individual synapses closely correlates with the number of AMPA receptors they contain [80, 81]. Besides the trafficking of AMPA receptors, other molecular mechanisms could be involved in the structural plasticity of dendritic spines. For instance, actin filaments play a crucial role in synaptic plasticity, given their propriety of changing the shape of the pre- and post-synaptic side and mediating the retraction and sprouting of synapses [82]. Accordingly, the pharmacological manipulation of actin polymerization and depolymerization effectively blocks LTP and simultaneously suppresses the structural enlargement of dendritic spines [83, 84]. Long-term plasticity and memory consolidation require, however, not only structural changes but also *de novo* protein synthesis. The gene expression pathway primarily implicated in these processes is activated by cyclic adenosine monophosphate (cAMP)-dependent mechanisms and mediated by members of the family known as cAMP-response element binding (CREB) proteins [85]. Specifically, cAMP activation initiates short-term synaptic changes that subsequently link—via nuclear translocation of protein kinase A and other kinases—to the activation and recruitment of CREB proteins and gene transcription [86]. Consistent with this assertion, overexpression of CREB promotes long-term memory storage from behavioral protocols that otherwise only induce short-term memory, indicating its pivotal role in the late phase of LTP and LTD [87, 88].

In addition to LTP and LTD, higher order dynamics have been shown to regulate synaptic plasticity, a phenomenon called metaplasticity [89]. The basic manifestation of metaplasticity is that the previous history of synaptic activity affects the direction and/or magnitude of activity-dependent synaptic plasticity in the future [90]. Notably, metaplasticity regulates synaptic plasticity across space and time (see for a review [91]). The time of metaplasticity expression typically spans from minutes to hours but can last for days and even weeks [92, 93]. Moreover, metaplasticity can be expressed over a broad range of spatial extents of the postsynaptic neuron. Indeed, heterosynaptic metaplasticity can be global, that is, cell-wide, or confined to specific dendritic compartments [94–96], whereas homosynaptic metaplasticity is only expressed at those synapses that participated in the initial bout of priming activity [97–99]. Metaplasticity phenomena have been originally described in the Bienenstock–Cooper–Munro (BCM) theory of bidirectional synaptic plasticity, which postulates that the synaptic modification threshold (*θ*_M_) is not stable but varies as a function of the integrated postsynaptic activity [100]. On one hand, *θ*_M_ decreases at a low level of previous postsynaptic activity, favoring the probability of subsequent LTP over LTD. On the other hand, *θ*_M_ increases at high levels of recent postsynaptic activity, thus favoring the induction of LTD over LTP [100]. Accordingly, in the hippocampus, repetitive activation of NMDA receptors can elicit a rapid shift in plasticity threshold such that LTP becomes difficult to elicit, and LTD induction is favored [101]. It has also been hypothesized that this sliding *θ*_M_ has an intrinsic homeostatic function, as it enables the maintenance of neuronal network activity within a physiological range [102]. Hebbian and homeostatic plasticity phenomena tend to interfere with each other if they are operating simultaneously at the same synapses [103]. Indeed, the latter is thought to represent a slow process that readjusts synaptic weights to stabilize circuit activity despite Hebbian plasticity [104, 105]. The induction of BCM-like metaplasticity involves multiple mechanisms, including NMDA receptor activation, and it is expressed by changes in NMDA receptor subunit composition [106].

All these synaptic plasticity phenomena occur constantly throughout the lifespan, although they appear influenced by age [107, 108]. In recent years, several tools, such as NIBS [109], have been proven effective in enhancing synaptic plasticity (and metaplasticity) even in mature brain structures and without significant external stimulation needed. In particular, NIBS has been shown to recruit activity-dependent synaptic plasticity and modulate both functional and structural plasticity processes [110–112]. For instance, high-frequency repetitive transcranial magnetic stimulation (TMS) protocols and theta-burst stimulation (TBS) induce LTP-like phenomena in the motor system of human subjects [113–115]. Similarly, transcranial direct current stimulation (tDCS) facilitates LTP induction and drives morphological changes at neuronal dendrites and spines in the animal model, thus suggesting its capability of enhancing plasticity in the human brain [116, 117]. Albeit we do not know if such interventions are effective on all brain circuits, it is plausible that NIBS can constructively interact with the natural processes of experience-dependent plasticity going on in the subject's brain during psychotherapy [118]. In the following paragraphs, we will outline the main neuromodulatory effects induced by psychotherapy and NIBS to explain how their action and combination could foster long-term learning and, therefore, enable consistent therapeutic changes.

## 3. Psychotherapy Effects on Neural Plasticity

According to the hypothesis put forward by Kandel [119], psychotherapy can produce enduring effects on human brain functioning and behavior, that is, the so-called therapeutic change. From a neurophysiological perspective, this process reflects long-lasting behavioral modifications achieved as a consequence of learning experiences involving the alteration of the strength and quality of synaptic connections [120]. Accordingly, neuroimaging studies conducted in the last decades have demonstrated that psychotherapeutic treatment produces durable changes in brain activity [3, 121–123]. Namely, investigations conducted on patients affected by major depressive disorder (MDD; [124–129]), anxiety disorders (ADs; [130–137]), obsessive–compulsive disorder (OCD; [138–143]), and posttraumatic stress disorder (PTSD; [144–148]) consistently highlighted psychotherapy-mediated modifications across several regions, including dorsolateral (dlPFC) and ventromedial (vmPFC) portions of the prefrontal cortex (PFC), cingulate cortex, insula, hippocampus, and amygdala. These brain areas, as well as the neuronal networks in which they are involved (e.g., limbic–cortical, prefrontal–striatal, and frontoparietal networks), play a pivotal role in the regulation of emotions, decision-making, and the encoding and retrieval of memories [149]. Among the most frequently observed changes in this research strand are the increased activity of prefrontal cortical areas (e.g., vmPFC and dlPFC [124, 130, 135, 136, 141]), the decreased activity of subcortical areas (e.g., amygdala and hippocampus [125, 129, 143]), and the enhanced functional connectivity between cortical and subcortical brain regions (e.g., vmPFC and amygdala [142]).

The aforementioned studies applied heterogeneous psychotherapeutic approaches, with cognitive–behavioral therapy (CBT) being the most employed. CBT is an evidence-based approach focusing on the interplay among patient's thoughts, feelings, and behaviors. Recognizing and understanding this interaction can help to modify maladaptive patterns and behaviors [150]. In particular, the cognitive model assumes that psychological disorders arise as a consequence of dysfunctional thinking [151]. Therefore, CBT aims first at recognizing and questioning patients' dysfunctional beliefs about themselves, the world, and other people and maladaptive coping strategies; and second to replace them with more functional beliefs and behaviors [150]. This process involves the application of techniques, such as daily logs, thought records, Socratic questioning, activity scheduling, graded task assignments, behavioral experiments, progressive relaxation training, and breathing exercises (see for review [152]). Due to its proven effectiveness, CBT is nowadays widely used across a variety of psychiatric disorders, including MDD, AD, OCD, eating disorders, and substance addiction [153]. Another psychotherapeutic approach widely applied in neuroimaging studies was prolonged exposure (PE), a specific type of CBT that teaches individuals to gradually approach their trauma-related memories, feelings, and situations instead of avoiding them [154]. This intervention is typically provided over a period of about 3 months with weekly individual sessions, encompassing the understanding of the patient's traumatic experiences, the explanation of treatment rationale, psychoeducation, and teaching of breathing techniques [154]. After that, imaginal and in vivo exposure can promote habituation to the feared stimuli [155]. To date, PE is strongly recommended for the treatment of PTSD [156].

Given the observed relationship between diminished vmPFC activity, heightened amygdala activation, and alterations in the autonomic nervous system and hypothalamic–pituitary–adrenal axis activity, a similar conceptualization has been proposed for PTSD [157, 158], AD [159], and MDD [160]. In the case of PTSD, psychotherapeutic treatment alters the amygdala–vmPFC–hippocampus network, which is highly involved in fear extinction [161], a pivotal learning mechanism characterized by a decline in conditioned fear responses following nonreinforced exposure to a feared conditioned stimulus. In such a circuit, the vmPFC and amygdala are involved in emotion regulation, while the hippocampus, responsible for furnishing contextual information to the amygdala, assumes a critical role in extinction learning [162]. Among exposure-based psychotherapies, PE facilitates extinction recall by enhancing connectivity among the vmPFC, amygdala, and hippocampus. Indeed, successful PE treatment in PTSD is associated with increased left dlPFC activity, downregulated amygdala activity, heightened hippocampal function during traumatic memory retrieval, and augmented vmPFC activity during fear-related stimuli processing [163, 164]. Another psychotherapeutic intervention able to sort long-lasting effects on the neural circuitry underlying PTSD is eye movement desensitization and reprocessing (EMDR). EMDR is a structured therapy that encourages the patient to focus on trauma-related memories while simultaneously experiencing bilateral stimulation to reduce the vividness and distress linked to those memories [165]. Indeed, the application of alternating bilateral sensory stimulation, that is, a visual EMDR protocol, provides a steep reduction in fear-related responses and yields sustained increases in the activities of the superior colliculus and mediodorsal thalamus [166, 167], attenuating the aftermath of traumatic memories.

Similarly, CBT induces heightened vmPFC, dlPFC, and vlPFC activation and diminished amygdala responsivity, leading to reduced depressive- and anxiety-related symptoms [168–173]. Efficacious psychotherapeutic interventions for MDD and AD elicit massive functional modifications within the limbic–cortical network [174, 175], which is involved in emotion regulation. Here, explicit modulation of emotions, that is, reappraisal processes involving a conscious endeavor to alter the interpretation of emotional stimuli, engages the dlPFC, dorsal anterior cingulate cortex (ACC), and parietal cortex [176]. Conversely, implicit emotion regulation, characterized by automatic processes devoid of introspective awareness, primarily hinges on the vlPFC and vmPFC [177, 178]. According to Mayberg's [174] model, psychotherapeutic approaches aimed at improving emotion regulation capacities enhance cognitive reappraisal mechanisms by augmenting top-down regulatory functions exerted by the PFC. Consequently, psychotherapy could fortify patients' adeptness in contending with negative affect, concomitantly regulating brain activity in regions involved in emotional processing (e.g., amygdala and vmPFC), thereby mitigating maladaptive information elaboration and attentional bias [179]. Notwithstanding considerable support, the proposition positing heightened top-down control of the PFC over limbic regions through psychotherapy does not comprehensively account for all clinical manifestations of these disorders. For instance, research indicates that nonconscious processing of fear in PTSD is correlated with amygdala and vmPFC hyperactivity, thus presenting a nuanced perspective [180]. In light of these considerations, psychotherapeutic interventions for PTSD that enhance vmPFC activity are expected to influence conscious threat processing but leave the impact on nonconscious threat processing uncertain, necessitating exploration in future targeted investigations.

Psychotherapy has also been shown to restore aberrant neurocognitive processes implicated in OCD, for example, cognitive inflexibility, compulsive behaviors, and compromised response inhibition, which correlated with the overactive ventral–striatal circuitry and diminished regulatory influence from the prefrontal–striatal and frontoparietal networks [181]. For example, CBT ameliorates pathological hyperactivity in cortico–striato–thalamic regions while concurrently reinforcing functional connectivity in other brain areas supporting compensatory processes, that is, insula and cerebellum [182–184]. Furthermore, brain activation in the right insula predicts CBT outcomes, with lower activation predicting a higher symptom reduction [185].

Overall, it is evident that psychotherapeutic interventions operate, at least partially, by augmenting prefrontal control over subcortical regions [121, 186]. However, following psychotherapeutic treatment, psychiatric symptoms face remission in approximately half of the cases [4], suggesting that such an intervention does not consistently restore aberrant cortical connectivity. Nonetheless, the investigations also demonstrate that psychotherapeutic intervention targets dysfunctional circuits in various pathologies. It is only in these areas that structural and functional alterations can be observed. This consideration opens the possibility of combining new approaches, such as NIBS, with psychotherapy to focally induce neural plasticity in impaired brain areas and, thus, develop effective treatments for refractory disorders.

## 4. NIBS: An Overview

NIBS has been widely used to modulate neuronal activity and synaptic transmission in adults [115, 117, 187], without resulting in relevant side effects for the recipient [11, 188]. Among NIBS, the two most frequently applied techniques are TMS and tDCS [189].

TMS consists of administering strong brief focal electromagnetic pulses through a coil placed over the participant's head that penetrates the skull to stimulate target brain areas [190]. The pulse generates suprathreshold neuronal membrane depolarization, inducing neuronal firing of cortical neurons beneath the coil [191]. TMS pulses can be applied through different stimulation protocols, such as single pulses TMS, which is typically applied in the clinical field as a diagnostic tool in neurological disorders (e.g., [192]), or as repetitive trains of stimulation, which can be either continuous at a specific frequency (rTMS) or patterned with specific interstimulus intervals, for example, intermittent theta-burst stimulation (iTBS) and continuous theta-burst stimulation (cTBS). Unlike single pulses TMS, rTMS, cTBS, and iTBS protocols are generally applied to induce long-term effects [193, 194]. Several stimulation parameters affect cortical activity, including frequency and intensity of the stimulation, shape of the magnetic pulse, duration of the stimulation period, and total number of pulses administered [195–197]. For instance, rTMS delivered at high frequencies (i.e., between 5 and 20 Hz and iTBS) causes excitatory effects and increases cortical excitability, whereas rTMS delivered at low frequencies (i.e., less than or equal to 1 Hz or cTBS) induces inhibitory effects and reduces cortical excitability [198, 199].

Conversely, tDCS involves delivering low-intensity electric currents through two (or more) rubber electrodes positioned in saline-soaked sponges or covered with conductive paste and placed over the scalp [200, 201]. One of the electrodes has a positive polarity (anode), and the other a negative (cathode). Unlike TMS, tDCS does not directly induce neuronal firing but modulates cortical excitability by a polarity-dependent shift of the neuronal membrane potential [202, 203]. The terminology anodal and cathodal tDCS reflect the placement of the anode and cathode electrodes over a target brain region [204]. Anodal tDCS is known to enhance cortical excitability, whereas cathodal tDCS has been shown to produce a reduction in cortical excitability [202, 203].

Although a detailed description of TMS and tDCS functioning goes beyond the aims of the current work, it seems crucial to highlight that NIBS outcomes in terms of cortical excitability and behavioral measures are difficult to predict in advance since they are the product of nonlinear interactions involving individuals' (anatomical, genetic, or hormonal) and contextual (functional, state-dependent, task, or activity-related) features, as well as stimulation parameters (frequency, polarity, intensity, number of sessions), leading to a high inter- and intra-individuals variability in NIBS responses [205–208]. Nonetheless, NIBS can yield significant cognitive and behavioral alterations [209–212] and reduced symptom severity among clinical populations. NIBS has been proven to be effective across many psychiatric disorders, for example, AD [213], MDD [214], OCD [215], PTSD [216, 217], eating disorders [218], and substance addiction [219, 220].

### 4.1. TMS Effects on Neural Plasticity

Converging evidence highlights that rTMS alters synaptic strength by facilitating LTP- and LTD-like mechanisms due to its ability to induce changes in cortical activity that exceed the period of pulse administration [114, 117, 221, 222]. Studies on corticospinal excitability suggested that high-frequency rTMS and iTBS applied over the primary motor cortex (M1) produce a long-lasting increase in motor-evoked potentials (MEPs) amplitude, indicating the occurrence of LTP phenomena and increased cortical excitability [223, 224]. Conversely, low-frequency rTMS and cTBS cause a decrease in the MEP amplitude, inducing, therefore, mechanisms comparable to LTD [113, 225]. A previous study suggests that the duration of rTMS-mediated plasticity effects depends on the number of pulses within the stimulation protocol [221]. Indeed, it has been illustrated that 150 pulses of rTMS at 5 Hz over the motor cortex do not produce major physiological changes, whereas the administration of 900 and 1800 pulses of rTMS at 5 Hz on the same brain site increases MEPs amplitude over 20 and 40 min, respectively [226]. These results are consistent with the outcomes of animal models, in which the persistence of in vivo LTP-like mechanisms could be prolonged up to days, depending on the number of stimulation trains applied to the rodents [227, 228].

Neuroimaging studies also reveal similar effects. Long-term modifications have been detected following rTMS in the cerebral blood flow and the blood oxygenation level-dependent (BOLD) signal [229, 230]. Furthermore, high-frequency rTMS applied over M1 and PFC results in long-term cerebral blood flow and glucose metabolism increase [231–233]. In contrast, applying cTBS over frontal eye fields causes a reduction in BOLD signal originating from such brain regions [229]. Why high-frequency rTMS causes opposite physiological effects than low-frequency rTMS is still poorly understood. Nevertheless, it has been hypothesized that NMDA receptors are massively involved in plasticity phenomena [221, 234], as animals exposed to neurostimulation exhibited increased expression of such proteins at the ventromedial thalamus, amygdala, and parietal cortex [235]. Theoretically, when postsynaptic neurons are sufficiently depolarized, NMDA cation channels open, allowing Ca^2+^ and Na^+^ to enter the cell, triggering signaling mechanisms that lead to long-term modifications in synaptic efficacy [236–238]. In light of these considerations, there is evidence that high-frequency rTMS causes a steep increase in calcium concentration in postsynaptic dendrites, augmenting the likelihood that presynaptic axon and postsynaptic dendrite are simultaneously active and, consequently, synapses become strengthened [239]. This means high-frequency rTMS triggers a rapid and strong increase in postsynaptic calcium levels, inducing LTP-like mechanisms [194]. Conversely, low-frequency rTMS causes a prolonged and moderate increase in postsynaptic calcium levels, inducing LTD-like mechanisms [240].

Several cellular mechanisms regulate rTMS-induced plasticity. The abovementioned synaptic events involving NMDA cation channels trigger long-term changes by remodeling dendritic spines and regulating neurotransmitter release [241, 242]. Nonsynaptic mechanisms could be involved as well, for example, neurotrophic factor release [243, 244], glial cell modulation [245], and epigenetic modifications [246]. Specifically, rTMS causes a significant increase in BDNF detectable in the hippocampus, parietal cortex, amygdala, and anterior parahippocampal gyrus [247, 248].

Despite significant advancements in understanding TMS mechanisms of action, much remains to learn about how these mechanisms relate to cognitive and behavioral effects observed in experimental subjects. Nevertheless, one established truth about TMS is that its effects are state-dependent, that is, the effects of TMS on brain networks are modulated by the initial activation state of the stimulated region [249]. The research strand on state dependency focused on various factors, including cognitive brain state, oscillatory brain state, and recent brain state history [250]. These states play a crucial role in determining the effects of TMS at the moment of stimulation, and thus, they are expected to be linked to clinical outcomes of TMS therapy [251].

### 4.2. tDCS Effects on Neural Plasticity

TDCS causes a wide range of consequences at a molecular level, which has been demonstrated by in vitro studies reporting that direct current stimulation of cortical and hippocampal sections induces relatively long-lasting forms of synaptic potentiation [116]. In most cases, tDCS results in significant alterations in the postsynaptic excitatory potentials (EPSPs) generated by the brain tissues [252–254]. These in vitro investigations have been conducted to unravel the cellular mechanisms triggered by tDCS and have contributed to the identification of two neuronal pathways that could underlie the behavioral changes induced by its application [255]. The first is the alteration of intracellular calcium concentrations. In this regard, an experiment conducted by Khatib, Golan, and Cho [256] unveiled that applying a direct current produces a significant increase in calcium concentration within the biological units under examination. Furthermore, such an increase is completely abolished by inhibiting phospholipase C, an enzyme triggering the release of Ca^2+^ from intracellular calcium stores, including the endoplasmic reticulum [257]. In the mechanism of action of tDCS, the release of calcium from intracellular stores appears, therefore, to be involved. The second pathway modulated by tDCS relates to the expression of BDNF. Indeed, applying a direct current to acute slices prepared from the mouse brain has induced massive changes in brain tissue [258]. Specifically, the stimulation results in a marked increase in BDNF levels and activation of associated tropomyosin receptor kinases, leading to the conclusion that anodal tDCS promotes the expression of BDNF [258]. Despite inconsistent findings (e.g., [259–261]), in vivo studies show that tDCS interferes with BDNF-related signaling pathways. A summary of the current evidence is provided by a meta-analysis which reveals that following the application of tDCS at the PFC and hippocampus, experimental groups show a marked increase in BDNF levels compared to controls [116].

Several studies on rodent models have been conducted over the years, showing that applying a direct current via surgically implanted electrodes causes an increase in neuroplasticity (e.g., [259, 261–265]). However, these investigations report mixed results depending on the stimulation polarity. For instance, some research shows that the application of anodal stimulation at the hippocampus results in enhancing LTP phenomena [262], whereas the use of cathodal stimulation over the same brain site produces no significant synaptic changes [263]. Yet, this is refuted by a previously mentioned in vitro study in which it has been demonstrated that the application of a direct current stimulation effectively modulates LTP at *cornus ammonis* hippocampal synapses, in a polarity-specific manner: anodal stimulation markedly increases LTP, whereas cathodal stimulation significantly reduces it [254]. Overall, among animal models, it is anodal, not cathodal, stimulation of prefrontal and hippocampal regions that produces a statistically significant enhancement of LTP phenomena compared to unstimulated specimens [116]. The absence of a significant promotion of LTP following the application of cathodal tDCS opens the hypothesis that the latter primarily modulates opposite forms of synaptic plasticity, namely LTD. This evidence is corroborated by an in vitro study conducted on neocortical sections taken from rats and humans in which a cathodal tDCS caused an increase in LTD-like phenomenon, that is, a marked decrease in the amplitude of EPSPs [266]. However, the literature suggests that cathodal tDCS selectively reduces LTP induction (e.g., [254]).

Additionally, synaptic changes induced by anodal tDCS are time-dependent. Indeed, in some cases, the first clear increases in LTP-like mechanisms are detected at a time immediately following treatment (i.e., within 60 min), while in others at a more prolonged time interval (i.e., after 60 min; [259, 262, 263, 265]). Nonetheless, the reasons why this occurs remain highly unknown and should be clarified in future research.

The effects of tDCS on neural plasticity entail changes in LTP induction and other synaptic modifications. As an example, Stafford et al. [267] observed a significant increase in AMPA receptor trafficking at the level of the hippocampus following the neurostimulation, while Jung et al. [268] demonstrated an increase in BDNF expression in synaptoneurosomes (an in vitro preparation of isolated synaptic boutons including both pre- and post-synaptic compartments) derived from the hippocampal area after its treatment with anodal tDCS.

Experiments on humans revealed that glutamatergic receptors, particularly NMDA channels, are involved in tDCS-induced modulation of plasticity mechanisms (e.g., [269–273]). Especially NMDA receptor-mediated calcium flux represents a critical component of synaptic plasticity, suggesting that intracellular dynamics concerning such ions are largely involved in the physiological effects of tDCS. It is also possible to conclude that stimulation-induced effects on neuroplasticity depend on both intracellular calcium levels and NMDA receptor responses and, therefore, that the cellular mechanisms triggered by the intervention are comparable to those observed during LTP and LTD of glutamatergic synapses [40]. In addition, Stagg et al. [274] highlighted that the application of tDCS produces a decrease in GABA concentrations at the cortical level, an effect that was also found to be highly correlated with the degree of impairment of glutamatergic activity detected in the same brain areas. A consequent hypothesis is that tDCS heals potential imbalances between inhibitory (i.e., GABAergic) and excitatory (i.e., glutamatergic) neurotransmitter systems [255, 275].

As for TMS, the effects of tDCS are state-dependent [276–278]. Indeed, cognitive changes occurring in healthy humans during both anodal tDCS and cathodal tDCS are affected by ongoing brain activity, increasing and decreasing, respectively, cortical excitability of task-related brain networks [207, 279]. In addition to the cognitive activity [280, 281], the oscillatory brain state also affects the effects of tDCS [282]. Such states contribute to the high variability of cerebral and behavioral responses to tDCS [208], which still limits its application for clinical purposes as monotherapy.

## 5. Toward an Integrated Approach

This manuscript focused on synaptic and neuronal modifications induced by psychotherapeutic treatment and NIBS application. These interventions have been associated not only with functional, physiological, and chemical changes but also with a decrease in the severity of patients' symptoms. However, only in a few cases psychotherapy and NIBS have been coupled, giving birth to a multimodal treatment aimed at achieving faster and longer lasting therapeutic changes [15, 16, 18]. Nonetheless, rTMS combined with CBT effectively improves the clinical conditions of patients affected by PTSD [283], AD [284], MDD [285], OCD [286], and substance addiction [287, 288]. Similar results have been observed when rTMS is coupled with PE, highlighting significant improvements in symptoms' severity reported by subjects with PTSD [289, 290], OCD [291], AD [292], and substance use disorders [293, 294]. Recently, rTMS has also been combined with mindfulness-based stress reduction (MBSR), highlighting a significant reduction in depressive symptoms' severity reported by subjects affected by poststroke depression [295]. TDCS has been coupled with psychotherapy as well, but currently, available evidence is highly heterogeneous. For instance, tDCS combined with cognitive training turned out to be effective in reducing craving among subjects with substance addiction in some cases [296], while it had no effect in others [297, 298]. Similarly, tDCS paired with cognitive psychotherapy results in clinical improvements in patients with MDD [299], but these results have not been confirmed by subsequent investigations [300]. Preliminary encouraging evidence on the efficacy of an integrated approach combining tDCS with psychotherapy has also been found for other psychiatric disorders, for example, PTSD [301], AD [302], and eating disorders [303].

In light of these data, we aim to provide a theoretical framework focusing on the rationale of combining psychotherapy and NIBS to boost therapeutic change. On one hand, psychotherapy could gain an advantage from NIBS application due to its ability to enhance synaptic plasticity phenomena recruited by cognitive and behavioral training and emotional processing. On the other hand, NIBS could benefit from psychotherapy because the latter might facilitate the efficacy of neuromodulation in a metaplastic manner. Additionally, neuronal modifications induced by psychotherapeutic interventions at specific or diffuse neural circuits could foster the following synaptic changes caused by NIBS, making them more likely and sustained.

We speculate that NIBS might play a pivotal role in enhancing metaplasticity, possibly enabling psychotherapy to induce further LTP- and LTD-like phenomena in the human brain. Indeed, consistent evidence for metaplasticity in the human cortex derives from NIBS investigations. For example, applying quadripulse stimulation with high-frequency priming over M1 causes a rightward shift of the threshold for the induction of LTP, that is, an enhanced probability of LTD-like plasticity and a decreased probability of LTP-like plasticity [304]. On the contrary, low-frequency stimulation priming causes the opposite effect, that is, a leftward shift of the threshold for the induction of LTP, suggesting an enhanced probability of LTP-like plasticity and a decreased probability of LTD-like plasticity [304]. Also, priming low-frequency rTMS over the motor cortex with high-frequency rTMS results in a stronger LTD-like MEPs decrease compared with low-frequency rTMS alone [305].

In this context, another important aspect relates to the metaplastic effects of neuromodulators and their role in psychiatric disorders. It is established that most forms of synaptic plasticity, including LTP, LTD, and spike-timing dependent plasticity, at almost all cortical and subcortical circuits (from the striatum to occipital and PFC), require the release of neuromodulators, such as dopamine and serotonin, at specific timing and dosages [306]. It was also shown that neuromodulator-dependent synaptic plasticity plays a role in the expression of psychopathological phenotypes, including depression and anxiety [307, 308]. Based on this, the observed changes in neuromodulator systems induced by psychotherapy (e.g., [309–311]) might further contribute to the efficacy of NIBS via a metaplastic route.

In addition, time-locking NIBS with a psychotherapeutic intervention can be a convenient approach, allowing clinicians to exploit the well-known state-dependent nature of NIBS. Namely, anodal tDCS elicits a widespread increase in cortical excitability at resting brain state [312], whereas cathodal tDCS fails to modulate cortical excitability, indistinguishable from sham stimulation [313]. Conversely, cathodal tDCS during a visuospatial attentional task reduced cortical excitability compared to the sham stimulation [279]. The possibility that the effects of neurostimulation depend on the brain state during stimulation is all the more significant. Consistently, in a study combining TMS and neuroimaging, it was observed that acute local and remote effects of TMS were influenced by the brain state during stimulation [314]. Since neurophysiological effects of neuromodulation could depend on the background cerebral activity, a pivotal question is how to combine NIBS with psychotherapy to maximize therapeutic effects. Unfortunately, when it comes to psychotherapeutic interventions, studies are typically heterogeneous considering the coupling of NIBS and psychotherapy, sometimes delivering neuromodulation before the psychotherapy session (as priming), sometimes during the psychotherapy session (as synergistic) and more rarely after the psychotherapy session (as consolidator) [15]. Hence, further investigations are needed to ascertain the optimal alignment between the two interventions.

## 6. A Transtheoretical Scheme for Implementing NIBS With Psychotherapy

During treatment, along with neuronal changes, cognition and behavior encounter the same fate, suggesting the existence of heterogeneous forms of plasticity that extend beyond the neural realm while remaining connected to it. In this framework, the notions of psychic and behavioral plasticity are introduced [315]. Psychic plasticity represents the ability to modify one's thoughts, evaluations, and beliefs, as well as to use endogenous and exogenous stimuli as input to modify one's point of view and to manage one's emotions functionally and adaptively [316, 317]. Psychic plasticity allows one to adapt to changes in context by modifying thoughts and emotions, thus enabling cognitive and emotional flexibility (i.e., the ability to shift attentional resources across environmental stimuli and manage one's emotional states according to experienced contingencies) [318, 319]. Behavioral plasticity refers instead to a change in one's behavior that results from exposure to internal or external stimuli, that is, when individuals are confronted with novel environmental conditions [320, 321]. Behavioral plasticity allows individuals to adapt to the context by changing their behavior [322, 323].

Increasing psychic plasticity represents one of the main goals of psychotherapy, followed by a gradual reduction of the patient's maladaptive coping strategies and behaviors. Conversely, NIBS promotes neural plasticity and modulates a patient's cognition and behavior [324, 325], facilitating therapeutic change. It is worth mentioning that the induction of greater neuronal plasticity does not necessarily correspond to improvements in cognitive and behavioral flexibility. Consistently, in our previous works, TMS treatment significantly ameliorated patients' symptom severity scores, but no significant cognitive enhancement was observed [326, 327]. These results support the claim that cognitive remediation interventions should be incorporated into the clinical pathway, as they might facilitate TMS-induced synaptic plasticity. In other words, if NIBS promotes the onset of synaptic plasticity phenomena, these would probably be consolidated following psychotherapy.

Furthermore, in psychopathology, it is highly plausible that neural, psychic, and behavioral plasticity are compromised [328]. For example, MDD is characterized by synaptic loss in dlPFC circuits that underlie affective and cognitive processes [329]. Neuroimaging studies consistently show lower brain volume in the dlPFC, ACC, and hippocampus in MDD patients [330] and reduced functional connectivity between PFC and limbic areas [331]. These neurophysiological deficits are accompanied by cognitive, emotional, and behavioral rigidity, such as tunnel vision [332], persistence of sad mood, anhedonia, and suicidal tendencies [333, 334]. Besides MDD, other psychiatric disorders are characterized by dendritic and synaptic pathology, including abnormal spine density and morphology, synapse loss, and aberrant synaptic signaling and plasticity [335]. For instance, patients with bipolar disorder and schizophrenia show a significant reduction in the number of spines per dendrite, decreased spine density, and diminished dendrite length compared to healthy controls [336, 337]. Evidence linking the dysregulation of synaptic plasticity to psychiatric disorders highlights the potential role of synaptic homeostasis in psychopathology, suggesting that such deficits likely represent a core pathophysiological feature of these conditions.

Hence, time-locking NIBS with psychotherapeutic interventions could be an effective approach to consistently promote neural, psychic, and behavioral plasticity simultaneously. As previously mentioned, therapeutic change involves not only behavioral modifications but also psychic ones, requiring, therefore, a major “firepower*”* to be achieved. In light of this consideration, we propose a transtheoretical scheme for coupling NIBS with psychotherapy, accounting for neural, psychic, and behavioral modifications that occur during treatment. In our scheme, NIBS, including TMS and tDCS application, primarily facilitates neural plasticity and indirectly affects behavioral plasticity, causing a symptom reduction in the relatively short term. In turn, psychotherapeutic interventions, such as CBT, PE, MBSR, and EMDR, directly promote behavioral and cognitive plasticity based on the specifics of the treatment protocol. Psychotherapy also exerts an indirect effect on neural plasticity in the long term. Metaplasticity and state dependency are pivotal in such an integrated approach since any neural, psychic, and behavioral change fosters further neural, psychic, and behavioral modifications. Also, considering the concept of synaptic homeostasis [338], the integration of psychotherapy with NIBS offers the advantage of physiologically “tuning” the plastic changes induced by neuromodulation through the contextual efficacy of the therapy. Such an approach could help prevent overstimulation of neural circuits beyond clinical necessity or the potential dissipation of the stimulation's ameliorative effects across broader brain networks.

Currently, our scheme is still a hypothesis that requires empirical validation, and it represents a mere simplification of the processes in place. Another limitation of our conceptualization is that it does not account for pharmacological interventions, which have been demonstrated to affect synaptic plasticity and, in turn, behavior, emotions, and cognition [339–341]. Moreover, we did not reserve the room for invasive stimulation strategies, for example, deep brain stimulation and epidural motor cortex stimulation, thus making our approach revisable for treating particularly refractory patients. Finally, although we argue that coupling NIBS and psychotherapy represents a potential approach for effectively counteracting psychiatric disorders, a gold-standard integrated protocol that facilitates plasticity induction and maximizes the effects of psychotherapy on patients' cognition and behavior has yet to be identified.

## 7. Potential Targets for an Integrated Approach

Given that both NIBS and psychotherapy modulate cortical activity, an integrated approach combining the two could be particularly effective in treating patients suffering from psychiatric disorders with etiologies characterized by cortical abnormalities. Activity in ACC and dlPFC has been shown to be altered in MDD, with some consensus that activity of limbic regions (i.e., ACC and amygdala) is increased, whereas in frontal regions is decreased [342]. Furthermore, structural neuroimaging studies show widespread cortical alterations in MDD patients as compared to controls, for example, adults with MDD have thinner cortical gray matter than healthy subjects in the orbitofrontal cortex (OFC), anterior and posterior cingulate cortex, insula, and temporal lobes [343]. Similarly, compared to controls, subjects with PTSD and AD display a hypoactivation of the vmPFC, OFC, and ACC, and a hyperactivation of the amygdala, indicating a failure of the extinction to fearful stimuli [344, 345]. Here, the implementation of a multimodal approach combining NIBS with psychotherapy has the potential to restore balance in the activity of frontal cortical regions, thereby positively influencing patients' symptomatology.

Aberrant functionality in cortical and subcortical brain regions has been also detected in OCD. Most notable are decreases in metabolism and perfusion of the ACC, caudate nucleus, thalamus, and multiple areas pertaining to the PFC, including OFC, dlPFC, vmPFC, and vlPFC [346]. Moreover, adults with OCD show a lower surface area for the transverse temporal cortex as well as a thinner inferior parietal cortex [347]. Thus, the application of integrated treatment protocols aimed at modulating the neural activity within regions of the cortico–striato–thalamo–cortical circuit could produce significant therapeutic effects in this clinical population.

Turing to eating disorders, anorexia nervosa (AN) has been associated with hyperactivity in brain regions involved in motivational and emotional processing, such as the amygdala, insula, and hypothalamus, alongside hypoactivation in brain areas related to inhibitory control, including the vlPFC, vmPFC, middle frontal gyrus, and both the anterior and posterior cingulate cortex [348]. In addition, AN patients display a decreased gray matter volume in the bilateral median cingulate cortex (extending to the bilateral anterior and posterior cingulate cortex) and left middle occipital gyrus (extending to the left inferior parietal lobe) [349]. Bulimia nervosa (BN) has been characterized by reduced activation in vlPFC and vmPFC [350], and heightened activation in the insula and striatum, reflecting disruptions in impulsivity and emotion regulation [351]. Notably, even subjects with subthreshold BN exhibit abnormalities of the dlPFC and OFC in gray matter volume [352]. Binge eating disorder has been linked to weaker activation of the PFC, which could be improved by CBT [353], and hyperactivity in temporal cortex and striatum, highlighting links to dissociative strategies and addictive behaviors [351]. Augmenting psychotherapy with NIBS to precisely act on the altered activation in these brain regions belonging to the fronto-striato and limbic circuits, which are theorized to play a pivotal role in the pathophysiology of eating disorders, could be a way to consistently ameliorate patients' clinical symptoms.

Functional neuroimaging studies on addictive behaviors have highlighted the key involvement of the PFC in modulating limbic reward regions and supporting higher order executive functions [354]. Impairments in PFC activity contribute not only to compulsive substance use but also to maladaptive behaviors characteristic of addiction, ultimately undermining autonomy and decision-making capacity [355]. Common to several addictive behavior is cue reactivity, in which exposure to stimuli induces craving as a consequence of Pavlovian conditioning [356]. Increased cue–reactivity converged in nicotine, alcohol, and cocaine addicts to the ACC, amygdala, and ventral striatum [357]. Moreover, a descriptive review of the literature suggests heightened activation in addicted individuals, particularly in prefrontal and orbitofrontal regions [358]. Cocaine addiction is associated with hyperactivity in the PFC, cingulate cortices, inferior frontal regions, and cerebellum during response inhibition, regardless of success [359]. Structural imaging reveals reduced volumes in the PFC, insula, inferior frontal gyrus, pregenual ACC, and anterior thalamus, with frontal gray matter declines correlating with addiction duration [360]. Furthermore, individuals with substance use disorders exhibit reduced cortical thickness in the insula and OFC compared to controls [361]. Therefore, addressing disturbances in frontostriatal circuitry through targeted modulation of PFC activity constitutes a preferential approach to alleviating symptom clusters specific to this clinical population, such as craving.

## 8. Conclusions

Considering that psychiatric disorders are characterized by the presence of pathologically altered neural plasticity and connectivity, combining neurostimulation with concurrent psychotherapy could restore aberrant synaptic plasticity along with inducing psychic and behavioral modifications. Indeed, several works suggested the convenience of time-locking NIBS with psychotherapy interventions to alleviate patients' symptomatology [15, 17, 19], although research in this field is still in its infancy. Future studies should better clarify the neural underpinnings of the pathological and curative processes to optimize protocol interventions and, in turn, treatment effectiveness. Further investigations could also focus on testing this integrated approach among clinical and subclinical populations by monitoring symptom severity trends and changes in psychic, behavioral, and neuronal functioning.

## Data Availability

Data sharing is not applicable, no new data are generated, or the article describes entirely theoretical research.
